# Integrative Pediatrics: Looking Forward

**DOI:** 10.3390/children2010063

**Published:** 2015-01-28

**Authors:** Hilary McClafferty

**Affiliations:** University of Arizona Center for Integrative Medicine Address: 3055 N. Campbell Ave. Suite 113, Tucson, AZ 85719, USA; E-Mail: hmcclafferty@email.arizona.edu; Tel.: +1-520-626-3503; Fax: +1-520-626-3518

**Keywords:** integrative medicine, pediatrics

## Abstract

Increase in the prevalence of disease and illness has dramatically altered the landscape of pediatrics. As a result, there is a demand for pediatricians with new skills and a sharper focus on preventative health. Patient demand and shifting pediatric illness patterns have accelerated research in the field of pediatric integrative medicine. This emerging field can be defined as healing-oriented medicine that considers the whole child, including all elements of lifestyle and family health. It is informed by evidence and carefully weighs all appropriate treatment options. This Special Issue of *Children*, containing a collection of articles written by expert clinicians, represents an important educational contribution to the field. The goal of the edition is to raise awareness about integrative topics with robust supporting evidence, and to identify areas where more research is needed.

Increase in the prevalence of diseases such as attention deficit disorder, obesity, diabetes, metabolic syndrome, autism, cancer, chronic pain, mental illness, asthma, and other inflammatory mediated illnesses has dramatically altered the landscape of pediatrics, impacting progressively younger age groups [1]. Subsequently, today’s children face serious health challenges, creating a demand for pediatricians with new skills and a sharper focus on preventive health. Limited treatment options, a desire to protect their child’s health, and a perceived lack of support from conventional clinicians have caused many parents to turn to complementary medicine. Widely published statistics indicate that an estimated 12% of children use complementary therapies, with prevalence increasing to 50% in children living with chronic illness. Use often occurs without disclosure to the clinician for fear of negative repercussions [2]. Patient demand and shifting pediatric illness patterns have accelerated research in the field of pediatric integrative medicine. This emerging field can be defined as healing oriented medicine that considers the whole child, including all elements of lifestyle and family health. It is informed by evidence and carefully weighs all appropriate treatment options.

Interest in integrative pediatrics has grown relatively quickly, in step with recommendations from the Institute of Medicine for increased education and training in the field to meet consumer demands for information on evidence-based complementary medicine [3]. The American Academy of Pediatrics (AAP) was an early supporter of the field, endorsing a Provisional Section on Complementary and Integrative Medicine in 2005. Full AAP Section status was earned in 2009 [4]. The name was simplified to the Section on Integrative Medicine (SOIM) in 2011, mirroring national streamlining of nomenclature in the field. Surveys of AAP members have indicated high interest in integrative pediatrics [5], yet, to date, relatively few educational opportunities exist. For example, a national survey of academic pediatric institutions in 2012 by Vohra, *et al.* [6] identified integrative pediatric programs at only 16 of 143 academic pediatric programs in 2012.

This special edition of *Children*, containing a collection of articles written by expert clinicians, represents an important educational contribution to the field. The goal of the edition is to raise awareness about integrative topics with robust supporting evidence, and to identify areas where more research is needed. Toxic stress, attention deficit disorder, vitamin D in children’s health, clinical hypnosis in children and adolescents, acupuncture for pediatric pain, integrative approaches to reflux, functional dyspepsia, and inflammatory bowel disease are some of the topics represented. Multiple case studies are included to demonstrate clinical application of integrative treatments.

Another development in the educational arena is the *Pediatric Integrative Medicine in Residency* (PIMR) program, a 100-hour online curriculum developed at the University of Arizona Center for Integrative Medicine. Designed to be embedded into conventional residency training, the program is halfway through a three-year national pilot phase at five US pediatric residencies: University of Arizona Department of Pediatrics, Stanford Children’s Health, University of Chicago Comer Children’s Hospital, Eastern Virginia Medical School/Children’s Hospital of the King’s Daughters, and the University of Kansas Department of Pediatrics, involving more than 300 pediatric residents and a dozen faculty. Early adopters of the program include Vanderbilt University School of Medicine Department of Pediatrics, Cardinal Glennon Children’s Medical Center, and the University of New Mexico Department of Pediatrics. The PIMR educational curriculum covers foundations of integrative medicine, including nutrition, mind-body medicine, physical activity, sleep, environment and health, and an introduction to whole medical systems. It also uses case-based teaching to introduce integrative approaches to common clinical conditions.

Although progress in the field of integrative pediatrics is evident, real obstacles exist, among them being unequal insurance reimbursement for children’s health issues [7,8], lack of research funding, skepticism from colleagues and administrators, compressed time during office visits, and competition from pharmaceutical companies. Recognition of these obstacles is important, yet so is acknowledgment of the relative lack of progress in prevention and treatment of complex pediatric conditions, such as obesity and autism, highlighting a call for new approaches. In reality, given the prevalence of integrative medicine use in children, even skeptics will require understanding of the potential risks and benefits of integrative treatments, in order to be prepared to direct patients and their families toward reliable resources.

It is possible that the most important benefit of integrative pediatrics is its potential for reduction in health care costs. Imagine the financial implications of a generation of children where the norm is healthy weight, mastery of self-regulation skills, avoidance of harmful environmental toxins, and the ability to apply evidence-based approaches to combat preventable chronic illness. Integrative pediatrics embraces each of these areas as fundamental to good health. In the search for models of medical care tailored to the children of today, I believe integrative medicine holds significant promise.

Hilary McClafferty, MD, FAAP
